# LncRNAs exert indispensable roles in orchestrating the interaction among diverse noncoding RNAs and enrich the regulatory network of plant growth and its adaptive environmental stress response

**DOI:** 10.1093/hr/uhad234

**Published:** 2023-11-17

**Authors:** Lingling Zhang, Tao Lin, Guoning Zhu, Bin Wu, Chunjiao Zhang, Hongliang Zhu

**Affiliations:** College of Food Science and Nutritional Engineering, China Agricultural University, Beijing, 100083, China; College of Horticulture, China Agricultural University, Beijing 100193, China; College of Food Science and Nutritional Engineering, China Agricultural University, Beijing, 100083, China; Institute of Agro-products Storage and Processing, Xinjiang Academy of Agricultural Science, Urumqi, Xinjiang 830091, China; Supervision, Inspection & Testing Center of Agricultural Products Quality, Ministry of Agriculture and Rural Affairs, Beijing 100083, China; College of Food Science and Nutritional Engineering, China Agricultural University, Beijing, 100083, China

## Abstract

With the advent of advanced sequencing technologies, non-coding RNAs (ncRNAs) are increasingly pivotal and play highly regulated roles in the modulation of diverse aspects of plant growth and stress response. This includes a spectrum of ncRNA classes, ranging from small RNAs to long non-coding RNAs (lncRNAs). Notably, among these, lncRNAs emerge as significant and intricate components within the broader ncRNA regulatory networks. Here, we categorize ncRNAs based on their length and structure into small RNAs, medium-sized ncRNAs, lncRNAs, and circle RNAs. Furthermore, the review delves into the detailed biosynthesis and origin of these ncRNAs. Subsequently, we emphasize the diverse regulatory mechanisms employed by lncRNAs that are located at various gene regions of coding genes, embodying promoters, 5’UTRs, introns, exons, and 3’UTR regions. Furthermore, we elucidate these regulatory modes through one or two concrete examples. Besides, lncRNAs have emerged as novel central components that participate in phase separation processes. Moreover, we illustrate the coordinated regulatory mechanisms among lncRNAs, miRNAs, and siRNAs with a particular emphasis on the central role of lncRNAs in serving as sponges, precursors, spliceosome, stabilization, scaffolds, or interaction factors to bridge interactions with other ncRNAs. The review also sheds light on the intriguing possibility that some ncRNAs may encode functional micropeptides. Therefore, the review underscores the emergent roles of ncRNAs as potent regulatory factors that significantly enrich the regulatory network governing plant growth, development, and responses to environmental stimuli. There are yet-to-be-discovered roles of ncRNAs waiting for us to explore.

## Introduction

As is widely recognized, only approximately 1.8% of the eukaryotic genome transcript is conventionally believed to contain protein-coding information. Traditionally, a substantial portion of transcripts were viewed as seemingly non-functional and were often regarded as transcriptional ‘garbage’ within eukaryotic organisms [1]. In the current era, propelled by the advancements in microarrays and high-throughput sequencing technology, there have been substantial portions of eukaryotic genomes transcribed. This transcription has brought forth an expanding spectrum of ncRNAs that play a pivotal part in orchestrating and regulating the intricate processes that underlie these vital aspects of plants, particularly in the context of plant growth, maturation, and their ability to adapt to external environmental stress. The emergence of ncRNAs substantially enriches the regulation network, where lncRNAs as prominent representatives have been reported extensively to exert pivotal and indispensable functions.

This review primarily focuses on plants to summarize our current understanding regarding the synergetic regulation of the ncRNAs. To begin with, we present a comprehensive classification of ncRNAs or own poor coding capacity RNAs based on their length, comprising small RNAs (18–30 nucleotides (nt)), medium-sized ncRNAs (31–200 nt), lncRNAs (>200 nt), and circle RNAs [2], and briefly provide synthetic sources of these various classes of ncRNAs. According to the findings from published research, it had been established that ncRNAs originate from a variety of sources, including gene breakage, intergenic area, transposons impact, insert of the genome, repetitive sequences and pseudogenes, and others [3]. Subsequently, the review delves into the coordination mechanism of ncRNAs and classic cases. Notably, the review also places a strong emphasis on the interaction relationship between small RNAs and lncRNAs. This interaction is of particular importance due to its capacity to induce potent regulation in the realms of plant development and environmental stress. Of course, other noteworthy regulatory mechanisms of lncRNAs are also illustrated. For instance, recent advancements in ribosome sequence have revealed the coding potential of lncRNAs for micropeptides, which is an intriguing facet. Finally, this review not only delineates the existing problems and challenges within the field but also provides forward-looking prospects and potential directions for further exploration.

## Small RNAs

**Figure 1:**
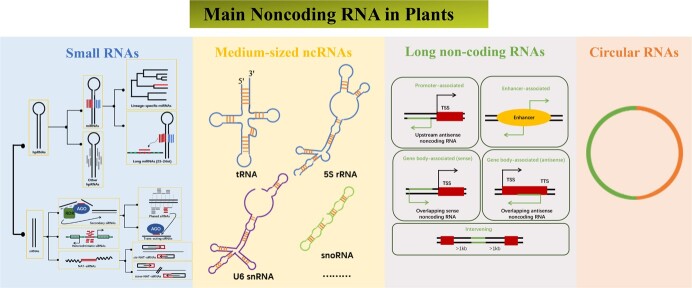
The classification of main ncRNAs in plants. **A**, the classification of small RNAs, the display from left to right represents hierarchical relationships, every solid box presents one type of small RNA. **B**, the classification of medium-sized ncRNAs. **C**, the classification of lncRNAs, the green line and arrow represent lncRNAs transcription direction while the black line and arrow represent coding genes. **D**, the diagrammatic sketch of circular RNAs. hpRNA, hairpin RNA; siRNA, small interfering RNA; miRNA, microRNA; NAT-siRNA, natural antisense transcript siRNA; phasi RNAs, phased small interfering RNAs; U6 snRNA, U6 small nuclear RNA; snoRNA, small nucleolar RNA; TSS, transcription start site; TTS, transcription termination site.

Here, the endogenous expressed small RNAs in plants originate from the cleavage action of the Dicer-like (DCL) protein and the binding of Argonaute protein [4]. Small RNAs have been documented that many classifications at different levels originate from the procedure of helical RNA precursors initially, including single-stand RNA precursors displaying a self-complementary ‘hairpin’ structure and double-strand RNA precursor (dsRNA) featuring an intermolecular hybridization structure. Therefore, at the primary level of classification, single-stand RNA precursors yield a category known as hairpin RNAs (hpRNAs) while dsRNAs generate small interfering RNAs (siRNAs). In the secondary distinction level, hpRNAs are further segregated into microRNAs (miRNAs) and other hpRNAs. siRNAs are mainly categorized into three types of secondary siRNAs, denoted as heterochromatic siRNAs, secondary siRNAs, and natural antisense transcript siRNAs (NAT-siRNAs). Within the tertiary level of categorization, miRNAs are divided into two subcategories, named lineage-specific miRNAs in minority species and long miRNAs (23–24 nt) [5]. Furthermore, secondary siRNAs are further subdivided into phased siRNAs or *trans*-acting siRNAs. NAT-siRNAs are divided into *cis*-NAT-siRNAs and *trans*-NAT-siRNAs. The primary focus of attention centers around miRNAs and siRNAs in current small RNA research [5] (Fig. 1A).

### miRNAs

**Figure 2:**
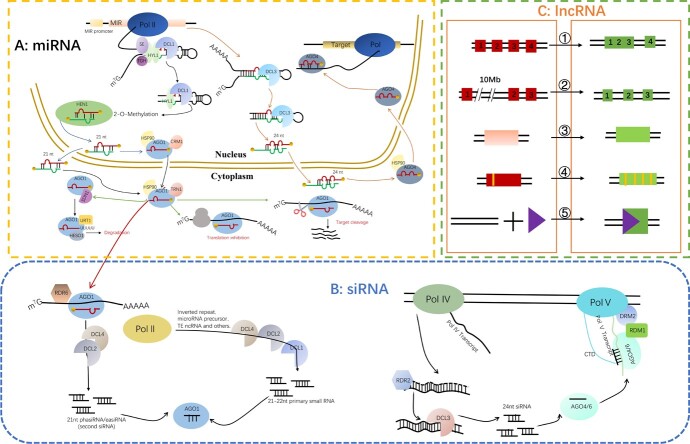
Source and production of miRNA, siRNA, and lncRNA. . **A**, the production diagram of miRNA, two solid lines separate the nucleus and cytoplasm. **B**, the production diagram of different lengths of siRNAs. **C**, the production source of lncRNAs, the red box represents the exon of coding genes, the green box represents the exon of lncRNAs, the triangle represents an insertion event, and the small grid represents a repeat event. (1) Frame disruptions of coding genes into ncRNAs; (2) Chromosome’ rearrangement; (3) Retrotransposition of non-coding genes; (4) Neighboring repeats within a ncRNAs; (5) Insertion of a transposable element. MIR, microRNA genes; SE, zinc finger protein SERRATE; TGH, G-patch domain tough; HYL1, HYPONASTIC LEAVES 1; DCL, RNase III family DICER-LIKE; AGO 1, ARGONAUTE 1; HEN1, methyltransferase HUA ENHANCER 1; HSP90, HEAT SHOCK PROTEIN90; TRN1, TRANSPORTIN 1; CRM1, CRM1/EXPORTIN1; SDN1, SMALL RNA DEGRADING NUCLEASE 1; HESO1, HEN1 SUPPRESSOR1. RDR, RNA-DEPENDENT RNA POLYMERASE 6; RDM1, DNA-binding protein; DRM2, DOMAINS REARRANGED METHYLTRANSFERASE 2.

miRNAs, as one of the principal small RNAs, ranging from 20- to 24-nt length, driving from highly precise excision of functional products, which usually own a well-defined set of mRNA targets. 21-nt miRNAs are prevalent in the majority of plant species. Besides, 22-nt miRNAs predominantly originate from foldback precursors that feature asymmetric bulges, only these 22-nt miRNAs possess the capability to initiate the emergence of RDR6-dependent siRNAs (RNA-dependent RNA polymerase (RDR)) from target RNAs in *Arabidopsi*s [6]. 23-nt miRNAs typically originate from DCL3 cleavage directly of the hairpin structure. Furthermore, the authors detected that 23-nt *miR156/157*, *miR164*, and *miR390* accumulated in members of the *Brassicaceae*, *Solanaceae*, and *Poaceae* families, respectively [7]. It is worth mentioning the existence of many 24-nt miRNAs within rice and *Arabidopsis* [8]. Moreover, the authors also reported 24-nt miRNAs *in vitro* plantlets of ponkan, the distribution of unique 24-nt miRNAs of ponkan leaves accounted for approximately 20.54%, followed by 20-nt miRNAs (22.15%) [9]. The biosynthesis process of miRNAs is a sophisticated and intricate regulatory process encompassing multiple pivotal stages, namely the transcription level, processing stage, modification step, and final assembly [10, 11]. In the transcription level, the microRNA genes (*MIRs)* are transcribed into stable pri-miRNAs with 5′ 7-methylguanosine cap and 3′-polyadenylated tail by DNA-dependent RNA POLYMERASE II (Pol II). In the processing stage, pri-miRNAs are first sliced into precursor miRNAs (pre-miRNAs) characterized by a stem-loop structure. Subsequently, pre-miRNAs are further processed into short miRNA/miRNA∗ duplex by an RNase III family DCL enzyme, which includes DCL1, or its homologous protein DCL2, DCL3, DCL4 [12]. These enzymatic activities are facilitated with the assistance of HYPONASTIC LEAVES 1 (HYL1) and zinc finger protein SERRATE (SE) and G-patch domain tough (TGH) or other factors (regulatory factor: CBP20/80, STA1, SIC, DBR1, GRP7, *et al*.). As a modification step, the miRNA/miRNA∗ duplex is methylated by 2’O-methylation on 3′ terminal ribosomes, which is catalyzed by the methyltransferase HUA ENHANCER 1 (HEN1) [13] and subsequently is degraded by 3′-5′ exonuclease (sRNA degrading nuclease1,2,3) [14]. In the final assembly, miRNAs are mainly assembled by AGO1 in the nucleus and then the miRNA∗ strand is exported to the cytoplasm to silence mRNA or inhibitof translation [15] (Fig. 2A). Furthermore, the intensity of miRNA-mediated inhibition is determined by polymorphisms within the hairpin precursors and the degree of complementarity between miRNAs and their targets [5, 16]. Additionally, there exists another category of miRNAs referred as to lineage-specific miRNAs. These miRNAs are characterized by their transient presence in the evolution history, lower expression levels, limited functionality, poor conservation, relative instability, and a lack of specific targets [5].

### siRNAs

siRNAs ranging in length from 21- to 25-nt, are double-stranded RNA molecules characterized by a specific chemical structure. The siRNAs can be detected in some homology-dependent RNA silencing events, often identified as the products of RNA degradation of both sense and antisense polarities [17]. In plants, most siRNAs are heterochromatic siRNAs that originate from repeat sequences and transposable elements (TEs) and are primarily responsible for silencing gene expression at the epigenetic level by promoting DNA methylation state and histone methylation activity of genes [5]. There are two different origins to generate siRNAs. One, referred to as RNA-directed DNA methylation (RdDM), contributes to forming 24-nt siRNAs. In this process, the chromatin remodeling factors CLASSY initially recruit Pol IV to bind to the RdDM loci site, which leads to generating single-strand siRNA precursors [13]. Subsequently, the precursors of siRNAs fold into double-stranded siRNA structures with the assistance of an RDR2. Finally, the immature double-stranded siRNA molecules are processed into mature siRNAs with the aid of DCL3. siRNAs typically bind to AGO4 protein to produce a significant effect mediated by Pol V. Parallel to this, Pol II contributes to an alternative biosynthesis pathway, named noncanonical RdDM. The pathway primarily produces 21- to 22-nt siRNAs, which are responsible for the initiation of DNA methylation [18] (Fig. 2B).

## Medium-sized ncRNAs

Certain members of noncoding RNAs are non-polyadenylated ncRNAs lacking poly (A) tails with lengths ranging from 50- to 300-nt, commonly referred to as im-ncRNAs. They have low expression levels that make them challenging to detect through experimental technology. Additionally, at the 5′ end of ncRNAs, possessing one phosphate, three phosphates, or trimethylguanosine cap may influence the stability, processing, and regulation of im-ncRNAs [19]. As illustrated in Fig. 1B, various types of im-ncRNAs are transcribed by Pol III, including tRNA, 5s rRNA, U6 snRNA, small nuclear RNA, 7SL/MRP (mitochondria recognition particle) RNA, NAT RNAs, and small nucleolar RNAs, which play roles in guiding ribosome modification [4]. Furthermore, Pol III contributes to the translation process by generating tRNA adaptor molecules that facilitate the transformation of mRNA codon information into amino acids [20]. Wang *et al.* revealed 838 im-ncRNAs and found an interesting phenomenon in which certain novel im-ncRNAs, derived from the 5’UTR of genes, consistently exhibited high expression levels [21]. The application of deep-sequencing technology is expected to discover more im-ncRNAs. It is worth noting that forthcoming research endeavors are anticipated to provide insights into the upstream regulatory factors and mechanisms of these im-ncRNAs.

## LncRNAs

LncRNAs are distinguishable from the small RNAs which own a determined base length and clear biological significance. However, lncRNAs typically arise through specific mechanisms and can be characterized using various criteria of their functionality as ncRNAs, their limited coding potential, and their significant nucleotide length (>200 nt) [22]. Similar to mRNAs, some lncRNAs also exhibit an m7G cap at the 5′ end and a poly-A tail at the 3′ end. These lncRNAs are transcribed by specific polymerases, such as PolI and Pol III. PolI, for instance, is predominantly associated with the transcription of genes containing lncRNAs within ribonucleoprotein complexes. It is responsible for transcribing some tandemly repeated genes into long rRNA precursors, then processing and assembling to form ribosomal subunits [22]. Furthermore, Pol III is primarily responsible for catalyzing relatively short (<500 nt) lncRNAs [22]. In addition, Pol II also produces a range of lncRNAs and pays some contributions to the lncRNAs’ production by ensuring the correct structure, accurate localization, and expression level [23]. Moreover, two plant-specific RNA polymerases, both of Pol IV and Pol V are involved in the generation of lncRNAs which can identify and repress TEs within the genome [24]. Based on the relative position between the lncRNAs and neighboring coding genes, lncRNAs can be categorized into five distinct classes, including promoter-associated lncRNAs (antisense ncRNAs mainly are upstream of coding gene), enhancer-associated lncRNAs (lncRNAs are associated with enhancer regions), gene body-associated lncRNAs (antisense and sense lncRNAs within the gene body of coding genes), and intervening associated lncRNAs (lncRNAs are positioned between two coding genes, lincRNAs) [22] (Fig. 1C). The origin of lncRNAs can be attributed in multiple ways. Firstly, lncRNAs may emerge through frame disruptions of coding genes, resulting in ncRNA sequences that retain some previous coding sequences. Secondly, non-transcribed genes or distanced sequence regions can give rise to multi-exon ncRNAs through chromosomal rearrangement. Thirdly, there is a retrotransposition function that makes some noncoding genes produce functional noncoding retrogene or non-function retropseudogene through duplication events. Fourthly, the presence of adjacent repeats of ncRNAs can lead to novel lncRNAs. Finally, the insert event of TEs gives rise to functional noncoding RNAs [25] (Fig. 2C).

## Circular RNAs

In addition to line RNAs, researchers also have unveiled the presence of thousands of endogenous circular RNAs (circRNAs) that are widespread in plants. Unlike linear mRNAs, circular RNAs formed a covalent closed-loop structure by splicing the RNAs’ head (5′ upstream acceptor) and tail (3′ downstream donor) and then combining them. The process, named back splicing, happens at post-transcriptional and co-transcriptional levels [26, 27]. The circRNAs are always considered an alternative form of pre-mRNA splicing [28] (Fig. 1D). The research indicated that circularization was more stable, enhancing their resistance to RNase R, and the half-life was more than 48 h *in vitro* when compared to linear RNAs [26]. Additionally, circRNAs are further divided into exon-intron circRNA (eIciRNA), exonic circRNA, intronic circRNA, and tRNA introns (tricRNA) based on gene location [29]. It has been acknowledged that plant circRNAs own the conservation characteristics. Researchers discovered that in *Oryza sativa* and *Arabidopsis*, the parent genes of over 700 exonic circRNAs exhibited as homologous [30]. Current research suggested the R-loop of circRNAs might modulate alternative splicing by binding miRNAs [31]. In *Arabidopsis*, an exonic circRNA originating from the *SPEALLATA3 (SEP3)* could form a DNA–RNA duplex with its parent genes to promote the event of the nascent transcript into the exon 6-skipped SEP3.3 isoform, finally contributing to the development of floral homeotic phenotypes [32]. In maize and rice, the research identified 149 differentially expressed circRNAs that responded to various environmental stresses such as heat, cold, or drought [33]. Furthermore, in tomatoes, overexpression of *PSY1-circ1* (involved in carotenoid biosynthesis) resulted in the yellow pericarp phenotype and *PDS-circ1* might regulate the expression of *PDS* mRNA to influence the color of plant organs [34]. Moreover, some lncRNAs can adopt circular RNAs and may compete with the linear pre-mRNAs for the recognition of splicing protein complexes, or as circular lncRNAs to sponge miRNAs [35].

## Coordinated regulation among ncRNAs

### LncRNAs play multiple roles

Based on the relative location of lncRNAs to coding genes, lncRNAs play multiple roles at multifaceted levels, encompassing the epigenetic, transcription, and post-transcription levels [22]. According to the orientation of transcription, the description is structured to cover aspects from the promoter regions to the termination of coding genes.

**Figure 3:**
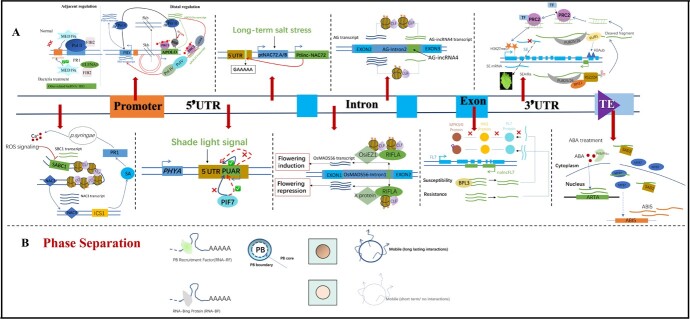
The classic regulation mode of lncRNAs in plants. **A**, The location of lncRNAs relative to coding genes and some classic regulation modes. The thick arrows indicate different examples involved in various mechanisms, the orange box represents the promoter region, the sky blue box represents the exon region, the green box represents lncRNAs, and the light blue box represents coding genes. The classic regulation modes of lncRNAs from promoter to termination site of the coding genes including promoter-related lncRNA *ELENA1* and *APOLO*, 5′ UTR-related lncRNA *PUAR* and *Ptlinc-NAC72,* intron-related lncRNA *AG-incRNA4* and *RIFLA*, exon-related lncRNA *nalncFL7,* the 3′ UTR -related lncRNA *SEAIRa*, TE-lincRNAs *ARTA* and other lncRNA *SABC1.***B**, The lncRNAs participating in phase separation. *PR1, pathogen response gene; HID, HIDDEN TREASURE 1;* MED19a, Mediator subunit 19a; PRC1, polycomb repressive complex 1; PRC2, polycomb repressive complex 2*; RDD, ROS1, DML2, and DML3, demethylases decrease; PHYA, PHYTOCHROMES; PIF7, PHYTOCHROMES INTERACTING; AG, AGAMOUS; CLF, CURLY LEAF;* SE, SERRATE protein*;* PUB25/26, plant U-box protein; RUB1, ubiquitin-like protein related to ubiquitin 1*;* RH11, RNA helicase; MPK3/6, MITOGEN-ACTIVATED PROTEIN KINASE 3/6; *HAI1, HIGHLY ABA-INDUCED PP2C1; FL7, FORKED-LIKE7;* BPL3, BPA1-LIKE PROTEIN3*; FIB2, FIBRILLARIN 2; SABC1, acid biogenesis controller 1; NAC3, transcription factor;* ICS1, isochorismate synthase 1*;* SA, salicylic acid*;* ABA, abscisic acid*;* SAD2, importin β-like protein*; MYB7, transcription factor; ABI5, the bZIP transcription factor;* PYR, pyrabactin resistance; PYL1, pyrabactin resistance 1-like.

To begin, the short promoter-related lncRNAs are typically generated within the promoter regions, which are caused by the early termination of gene transcription by Pol II [36]. In contrast, lncRNAs would either directly or indirectly regulate promoter activity. For example, in plant immunity, lncRNA *ELENA1* possessed the capability to dissociate the FIB2/MED19a protein complex and released the FIB2 protein from its repressive influence on the *PR1* promoter [37]. Furthermore, lncRNAs not only regulate adjacent genes in transcription levels but also affect distal genes by bringing them into close spatial proximity, contributing to forming a three-dimensional genome organization in *trans*. As an example, a distant lncRNA, such as *APOLO*, was typically derived from a genomic locus located approximately 5 kb upstream of the gene *PID*. There was a chromosome loop formed between the *APOLO* and the *PID* promoter, mainly by recruiting epigenetic marks like histone H3 lysine 27 trimethylation (H3K27me3) and DNA methylation, which were instrumental in the regulation of auxin transport in *Arabidopsis* [38]. Moreover, the lncRNA *APOLO* was also substantiated to interact with the transcription factor *WRKY42* and then modulate the binding of *WRKY42* to the promoter of *ROOT HAIR DEFECTIVE 6* (*RHD6*), ultimately triggering root hair cell expansion in response to cold stress in *Arabidopsis* [39]. Furthermore, lncRNA would establish an intragenic gene loop. For instance, the lncRNA *COLDWRAP* was transcribed from the locus between the promoter and first intron of *FLOWERING LOCUS C (FLC)* which played a pivotal regulation role in flowering time during *Arabidopsis* vernalization [40]. Furthermore, some lncRNAs originate from the promoters of TEs and can influence gene promoters by RdDM at the edges of TEs [41]. Likewise, during the photomorphogenesis process under contributing red light exposure, another promoter-associated lncRNA *HIDDEN TREASURE 1* (*HID1)* was reported as a composition of the large nuclear ribonucleoprotein complexes to repress the transcription level of P*HYTOCHROMEINTERACTING FACTOR 3,* consequently enhancing the photomorphogenesis process [42] (Fig. 3A).

Secondly, the 5′ UTR refers to the genomic interval between TSS where Pol II initiates transcription and the AUG start codon of the associated mRNAs. An update reported that a lncRNA related to the 5′ UTR regions, named *PUAR* (*PHYTOCHROMES* (*PHYA*) UTR antisense RNA), is involved in the plants’ shade avoidance syndrome (SAS) phenomenon, which helps plants get more light by initiating a sequence of morphological and physiological changes. LncRNA *PUAR* was primarily induced by plant shade areas, then reduced the accumulation of *PHYA,* and finally enhanced shade-induced hypocotyl elongation. Furthermore, the researchers also discovered that *PUAR* would block a positive regulator, *PHYTOCHROMES INTERACTING* (*PIF7),* which was binding to the 5′ UTR of P*HYA* [43]. Under salt stress conditions, another 5′ UTR-related lncRNA was capable of binding to a specific motif within 5′ UTR not coming from the 5′ UTR region. An example of this was *Ptlinc-NAC72* upregulated *ptNAC72.A/B* by identifying a tandem element (GAAAAA) of the 5′ UTR of two p*tNAC72.A/B* genes in *Populus trichocarpa* [44]. Furthermore, in *Arabidopsis*, the DCL4 gene utilized lncRNAs to influence TSS selection and gene sequence. The mutant of missing lncRNA of *DCL4* would reduce the DNA methylation and alter the selection of the gene TSS locations by Pol II [45].

Thirdly, some intron regions can produce lncRNAs, as an example, *AG-incRNA4*, one transcript originating from the second intron of *AG (AGAMOUS)*, which encodes a MADS-box protein involved in stamen and carpel fates of *Arabidopsis* flowers. The *AG-incRNA4* knockdown mutant reduced the recruitment ability of PRC and decreased the H3K27me3 level within the *AG* gene body to accumulate the *AG* mRNA level [46]. Some intron-derived lncRNAs act as components of complexes to exert influence. For instance, *RIFLA (RICE FLOWERING ASSOCIATED)* was transcribed from the first intron of *OsMADSD56 (MIKC-type MADS-box protein 56)*, and it was believed that *RIFLA* might interact with *osiEZ1* (one gene related histone H3K27-specific methyltransferase) to form a complex and then reduced *OsMADSD56* expression to regulate the flowering of *O. sativa L* [47] (Fig. 3A). Furthermore, the exon-related lncRNAs had been found within the *TFIIIA* gene and participated in the synthesis process of Pol III GTF TFIIIA. A noncoding 5S rRNA structural mimic (P5SM), as the second isoform, mainly bound L5 ribosomal protein, hereby promoted the synthesis of Pol III. Additionally, *P5SM* was instrumental in maintaining the levels of L5 protein and 5S rRNA [48]. Moreover, it had been reported that *nalncFL7* was one transcript derived from the antisense strand of *FORKED-LIKE7* (*FL7*, At4g060410), which overlapped with exon 2–7 of the *FL7* locus in *Arabidopsis*. Research findings further revealed that the *nalncFL7* transcript bound BPL3 (an RNA binding protein) and inhabited the transcript level of *FL7*, further regulated HIGHLY ABA-INDUCED PP2C1 (HA1)-mediated MPK3/6 dephosphorylation, and ultimately participated in the immunity reaction triggered by *Phytophthora capsicum* [49] (Fig. 3A).

Fourthly, some intragenic lncRNAs that are derived from the 3′ end of coding genes would participate in the RNA process. As an example, an antisense intragenic lncRNA *SEAIRa* originated from the 3′ end of the *SERRATE (SE)* gene and downregulated the expression level of *SE* to regulate serrated leaves of *Arabidopsis. SEAIRa* accomplished the process by recruiting plant U-box protein PUB25/26 and a ubiquitin-like protein related to ubiquitin 1(RUB1) for H2Aub, resulting in recruiting H3K27me3 marks, which accumulated at the first exon of *SE* [50]. (Fig. 3A).

Furthermore, some lncRNAs originate from the sequence area of transcription terminators of coding genes due to a failure in terminating transcription, and many of these are antisense lncRNAs [51]. For example, one cold-related lncRNA *SVALKA* was triggered from a neighboring downstream gene and participated in a cold tolerance event mediated by the *C repeat/dehydration-responsive (CBF1)* transcription factor. *SVALKA* had interacted with a cryptic lncRNA *asCBF1* which came from the antisense strand of the overlapping *CBF1*. The cascade of *SVALKA* and *asCBF1* modulated the cold acclimation process of the plants by affecting the expression level of *CBF1* mRNA [52]. The opinion on the relationship between transcription termination and lncRNA production is centered on the following aspects. Transcription termination itself likely regulates the activity of the antisense transcript promoters and controls lncRNA production [22]. In addition, lncRNAs would regulate the initiation of antisense transcription by forming R-loops [53]. What is more, the genomic regions located beyond the transcription terminator would affect the expression of the coding gene; as an example, lncRNA *SUF* in male identification of plants [54].

Lastly, in addition to the lncRNAs that are directly related to certain genes, there are also some lincRNAs or lncRNAs from other origins that play crucial roles in responding to environmental stimuli and regulating plant growth. For instance, lincRNA *SABC1* acted to suppress a transcription factor *NAC3* in *cis* and subsequently triggered the expression of the salicylic acid (SA) biosynthesis enzyme, named isochorismate synthase 1 (ICS1), leading to a dampened immunity response and promoting healthy plant growth [55]. Furthermore, some lincRNAs exert effort in plant development and ripening. Zhu and their teams focused on the roles of lncRNAs in tomatoes which serve as typical respiratory climacteric model plants [56]. They first identified 3679 lncRNAs (approximately 85.1%) belonging to lincRNAs types related to tomato ripening. Moreover, in classical ripening mutants *rin* (*RIPENING INHIBITOR*), they also found 490 up-regulated lncRNAs and 187 down-regulated lncRNAs [56]. The subsequent research revealed that 187 RIN-targeted lncRNAs had been identified as having a RIN binding site in their promoter regions. Particularly, one lncRNA among them, named *lncRNA2155,* had been shown to have a delaying effect on tomato fruit ripening in *vivo* and in *vitro* [57]. Additionally, another paper cloned the full length of *lncRNA1459,* and in the loss-of-function mutant of *lncRNA1459,* ethylene production, and lycopene accumulation, two key factors in fruit ripening, were notably inhibited [58]. Some lncRNAs come from TEs, named TE-lincRNAs. A TE-lincRNAs *ARTA* mainly bound to the carboxyl-terminal area of an import β-like protein known as SAD2, and then inhibited the entry of the transcription factor *MYB7* into the nucleus to free the inhibition of *MYB7* to *ABI5* (the bZIP transcription factor). Moreover, the regulation process was induced by abscisic acid (ABA). Under ABA treatment, the regulation led to an accumulation of *AR* expression and then positively regulated the expression level of *ABI5*, thereby reducing drought tolerance in *Arabidopsis* [59] (Fig. 3A). During the early endosperm development of *O. sativa*, there was a parent-of-origin lncRNA *MISSEN *that played a role in hindering the function of the helicase family protein (HeFP). This interference affected the expression of the tubulin gene and resulted in the abnormal aggregation of the cytoskeleton. As a consequence, this disruption in the cytoskeletal organization led to obvious dents and protrusions on seeds [60]. Furthermore, some lncRNAs exert a significant influence on alternative splicing (AS) events; for example, lncRNA *ACoS-AS1* participated in the *trans*-splicing between *SlPsy1* (regulation enzyme in carotenoids biosynthesis pathway) and *ACoS-AS1,* resulting in a yellow tomato fruits phenotype [61]. Other research teams found that 72.55% of lncRNAs caused AS in different tomato tissues, including the root, leaf, and flower. Particularly, during the initial flowering time of tomato, which yielded a range of 16 995 AS events, among the various types of them, ranked first is alternative first exon (AFE), followed by a retained intron (RI) events [62]. Furthermore, lncRNA *ASCO* contributed to the assembly of key splicing composition PREMRNA PROCESSING 8 (PRP8) and SmD1, which influenced the binding of PRP8 to a subset of its pre-mRNA targets in *Arabidopsis* [63]. Researchers reported that the combination of factors of methyl jasmonate (MeJA) treatment and transcription factor *NtMYC2* would also regulate the expression levels of several lncRNAs through qPCR detection and gene editing technology [64].

### LncRNAs may participate in phase separation

Phase separation is a newly emerging phenomenon that introduced an additional regulatory mechanism in cells in response to environmental stimuli. In countless pieces of research, phase separation has been recognized as the basis or a contributing factor to the formation of biomolecular condensates [65]. Correspondingly, in the cellular microenvironment, the biomolecular condensates typically represent membrane-less compartments comprised of non-stoichiometric assemblies of proteins or nucleic acids [66]. The condensates mainly encompass two molecular complexes: one type is stress granules (SG) which consist of a dense core containing all pivotal components and a peripheral shell that serves a sequestration function. These condensates often develop major responses to environmental changes through a conserved transient mechanism. Another type of biomolecular condensate is referred to as the process body (P-body), which is independent of stress conditions [66]. Nevertheless, it is worth noting that there is limited evidence regarding whether lncRNAs were SG-enriched RNA species due to the poor detection technology. However, lncRNAs were also core components of recruiters. For example, under oxidative stress, some lncRNAs had been detected in cells. Moreover, those lncRNAs accumulated in SGs tend to have lower cellular expression levels than those missing in SGs [67]. It is well-established that lncRNAs exhibited tissue or cell-type specificity and low expression levels compared to other ncRNAs. Furthermore, there was evidence to suggest that the interaction between lncRNAs and P-bodies or SGs is relatively short-lived [68, 69]. Moreover, the lncRNAs in P-bodies or SGs are easy to overlook or not observed because of limited approaches. Hence, we look forward to more methods being developed. The related views had been reported in animal cells that lncRNAs were reported as elements of SGs. There are around 60% of lncRNAs transcripts localized to SGs during the DNA damage process [67] (Fig. 3B).

### Coordinated regulation between lncRNA-miRNA

**Figure 4:**
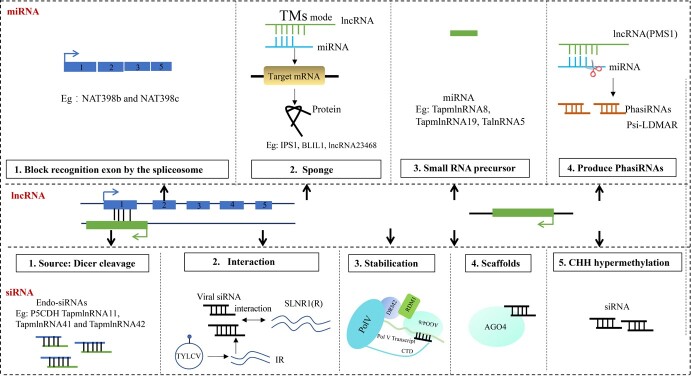
The coordinated regulation among ncRNAs (miRNAs and siRNAs) and lncRNAs. The upper diagram shows lncRNAs as a blocking factor, a precursor, and a sponge for miRNAs. The lower diagram shows the production origin of siRNAs from lncRNAs CHH hypermethylation and dicer cleavage, or lncRNAs as a stabilization factor of the transcription complex, as scaffolds to bind the AGO4-siRNA complex to exert a regulation effect. TYLCV, tomato yellow leaf curl virus.

Usually, the formation of lncRNA–RNA duplexes is associated with post-transcriptional regulation, where lncRNAs interact with other RNA molecules to regulate the coding gene expression. Moreover, in plants, the form of lncRNAs are *cis* NATs, which mainly affect gene silencing or translational promotion [70]. For example, approximately 70% of annotated mRNAs of *Arabidopsis* species were associated with detectable lncNATs [42]. These lncRNAs always play various roles in the regulatory network between lncRNAs and miRNAs, serve as a source of miRNAs, act as bait and sponge to sequester miRNAs, or interfere with the precise cleavage process of pri-RNA, and, in turn, influence the target mRNAs [71]. For example, lncRNA *NAT398b* and *NAT398c* were in co-expression with *MIR398b* and *MIR398c* in *Arabidopsis*, respectively. Overexpression of lncRNA *NAT398b* and *NAT398c* negatively regulated the biosynthesis of *miR398* by destabilizing the *pri-miR398b/c*, leading to the upregulation of miR398-targeted genes *CSD1/2/3* and *CCS.* These target genes primarily participated in processes related to cell death, oxidative stress and heat stress, ultimately affecting the thermotolerance of *Arabidopsis* [72]. In plants, lncRNAs also known as ‘target mimics’ (TMs), could function as competing endogenous RNAs (ceRNAs). These lncRNAs owned quite similar target binding sites of miRNAs (some miRNA recognition sequences) within incomplete base pairing. Moreover, these lncRNAs impaired the activity of the miRNAs and blocked the miRNAs binding to their authentic target transcripts [73]. A classic example of this competitive mechanism in plants was the noncoding gene *IPS1 (INDUCED BY PHOSPHATE STARVATION1)*, which owned a conserved 23-nt-long motif with sequence complementarity with *miR399*. Thus, *IPS1* would bind *miR399* by forming a mismatched loop to the accumulation of the target mRNA *PHO2*, further resulting in lower inorganic phosphate (Pi) content and reduced Pi remobilization in the shoot, which was first reported in *Arabidopsis* [74]. Furthermore, in a study by Hou *et al*, *lncRNA39026* was identified as an endogenous target-mimicry for miR168a. The interaction enhances tomato resistance to *Phytophthora infestans* by inducing the expression of PR genes [75]. In response to blue light stress and mannitol stress, the researchers revealed the existence of blue light-induced lncRNA *BLIL1.* lncRNA *BLIL1* competed with *miRNA167* and influenced the target mRNA *ARF6/8.* Hence, the authors put forward the *BLIL1-miRNA167-ARF6/8* regulation network in the hypocotyl elongation in *Arabidopsis* [76]. Additionally, in the context of the plant resistance process, silencing specific *lncRNA23468* led to the accumulation of the expression level of *miR482b* and resulted in reduced levels of the target gene *NBS-LRRs. NBS-LRRs* gene mainly took part in resistance of *phytophthora* infestation in tomatoes [77]. Several reports have presented that certain lncRNAs can serve as precursors for miRNAs through intracellular cleavage activities. One study described a pair of lncRNAs derived from cotton subgenomes that could generate *miRNA397.* Furthermore, *miRNA397* mainly repressed the expression level of *LAC4* by guiding mRNA degradation. The process involved the regulatory process of lignin metabolism and the domestication of tetraploid cotton fibers [78]. Furthermore, an additional research team discovered that *miRNA397* down-regulated its target laccase-like gene transcripts in rice [79]. Furthermore, the investigators presented compelling evidence that certain stress-related transcripts, named *TapmlnRNA8*, *TapmlnRNA19*, and *TalnRNA5* owned stable hairpin structures and served as precursors for miRNAs during powdery mildew infection in wheat by mapping miRNA technology. Among these, two lncRNAs (*TalnRNA5* and *TapmlnRNA19)* and *TapmlnRNA8* emerged as the precursors of *miR2004* and *miR2066,* respectively. Additionally, within the same context of publication, the authors also identified a lncRNA, denoted *TahlnRNA27*, which possessed a sequence from the *Ta-miR2010* gene family and was notably up-regulated in ‘TAM107’ cultivar (a heat tolerant wheat cultivar) after heat treatment [80] (Fig. 4).

### The coordinated regulation between lncRNA-siRNA

Plant-specific Pol IV and Pol V have been conventionally recognized as key enzymes in the production process of 24-nt siRNAs [24]. Furthermore, lncRNAs transcribed by Pol V have been observed to facilitate the recruitment of silencing machinery compositions to the gene promoter region, thereby affecting repression of gene expression [81] and impeding the read-though of the genes [82]. Some ncRNAs can create double-stranded RNA structures through binding to NATs. This interaction serves as the foundation for generating siRNAs, including lncRNAs, thus lncRNAs as a source of siRNAs. Within this context, two NATs molecules, Delta(1)-pyrroline-5-carboxylate dehydrogenase (*P5CDH*) and an unidentified gene, *SRO5*, had been identified as responsible for generating 21-nt siRNA by DCL1. The coordination process regulated salt tolerance in *Arabidopsis* [83]. A research team identified that the stress-responsive lncRNAs also served as the origin of siRNAs in *cassava* [84]. Moreover, when subject to cold treatment, the researchers observed that approximately 18.34% of lncNATs might potentially serve as precursors for the ranging in 19–25 nt of siRNAs. For example, *lncNAT14-Manes.05G207400* and *lncNAT179-Manes.14G040500* produced a significant number of 2127 siRNAs through mapping these siRNAs reads to the overlap region of the two lncNATs [85]. Recently, a novel model describing the interaction between siRNAs and their host lncRNAs came to light. This model meant that the generation of siRNAs transcribed from lncNATs might regulate the expression of their corresponding complementary sense strands. In the context of resistance against TYLCV, researchers found that the viral siRNA (vsRNA) generated from the 25-nt noncoding intergenic region (IR), which displayed a near-perfect complementary with a lncRNA *slLNR in* TYLCV-susceptible tomato cultivars. Furthermore, they also found the expression of vsRNAs would induce silencing *slLNR1*, which was related to the manifestation of curled leaf and stunted plant phenotypes, highly akin to TYLCV symptoms [86]. Under heat stress and powdery mildew infection in a wheat cultivar, both *TalnRNA9* and *TalnRNA12* were identified as variants of *signal recognition particle (SRP) 7S RNA*. Additionally, *TapmlnRNA11*, *TapmlnRNA41*, and *TapmlnRNA42* were observed to be modulated by 24-nt siRNAs, encompassing five groups that exhibited matching with both *TalnRNA9* and *TalnRNA12*. Furthermore, when considering different susceptible and resistant wheat cultivars, the act of inoculation with powdery mildew was found to increase the expression level of *TalnRNA9* and *TalnRNA12* and concurrently decrease the abundance of the antisense sequence [80]. It is important to note that in the model plant *Arabidopsis*, an intricate process was referred to as the RNA-mediated transcriptional gene silencing pathway or RdDM [87]. The key factors of the process encompassed lncRNA, siRNA, and conserved AGO [81]. Specifically, siRNAs produced from PoI IV, RDR2, and DCL3 were responsible for binding ARGONAUTE 4 (AGO4). This interaction served to establish sequence specificity via base-pair complementation at specific loci [88]. The acquisition of lncRNAs mediated by PoI II and plant-specific PoI V served as scaffolds for binding the AGO4-siRNA complex and directing targeted AGO4 to specific genomic loci [89]. Typically, the lncRNAs derived from Pol V collaborated with siRNAs to facilitate the locus-specific establishment of RdDM [90]. Some lncRNAs are associated with epigenetic regulation, especially the DNA methylation of cytosine [91]. Researchers had highlighted that in a null mutant of *OsMET1–2^−/−^*, a DNA methyltransferase 1 in rice, the TEs family En/Spm experienced transcriptional de-repression because of the genome-wide erasure of CG methylation. This phenomenon led to the production of plenty of specific lncRNAs [92]. Additionally, there was an accumulation of RdDM-mediated CHH hypermethylation in the 5′-upstream genomic regions of lncRNAs. Furthermore, a substantial number of siRNAs and distinctive hypermethylated regions were found to co-localize by sRNA sequence technology. These regions were related to both common and mutant-specific lincRNAs [92, 93]. For instance, among them, 61.49% and 74.53% exhibited CHH hypermethylation and were enriched with siRNAs in their 5′-upstream region, respectively. Additionally, it was obverted that the simultaneous presence of CHH hypermethylation and the higher abundance of siRNAs was found to be approximately 52.17%. These findings suggested that siRNAs may take part in the regulation of lncRNA expression by DNA methylation [92]. Furthermore, the paper uncovered that the transcription of Pol V was diminished in mutants lacking key components of the RdDM. The observation implied the existence of a positive feedback loop between DNA methylation and non-coding transcription, which served to reinforce the process of transcriptional silencing [90] (Fig. 4).

### Coordinated regulation of other RNAs

Authors had reported that circRNAs acting as ceRNAs exert a regulated role in the expression level of miRNAs by absorbing and competitively suppressing the activity of miRNAs within plant systems. For example, the mechanism was exemplified in the diR156-related circRNA-miRNA-mRNA network in *Arabidopsis* through establishing and analyzing by comprehensive investigations of the PlantCircNet. Within this network, certain circRNAs and mRNAs had been predicted to serve as targets of *miRNA156a-5p*. Moreover, within the network, *AT1G27370*, *AT1G53160*, *AT3G15270,* and *AT5G43270* had been experimentally validated [94]. Furthermore, the paper provided another regulatory network about a Heat Shock Protein 18.2 (HSP18.2, AT5G59720), regarded as *circRNAs–miR414–AT5G59720* in *Arabidopsis*. As per the findings, *HSP18.2* could generate 19 circRNAs, which were targeted by *miR414,* the only miRNA in this network [94]. As we all know, *miRNA414* exhibited a high degree of conservation and assumed crucial roles in various processes throughout plant growth and development. Particularly, the significance is pronounced in response to alterations in environmental conditions, such as high or low temperature, oxygen concentration and irradiation intensity [95, 96]. Also, circRNA *ATH_circ09039* was predictively correlated to light-related (PRJNA218215) and stress-related (PRJNA213635) samples in this regulatory network [94]. Moreover, the plant circRNAs serve as miRNA sponges, for example, in tomatoes, 163 circRNAs had demonstrated a chilling response. Among these, 102 circRNAs were predicted to contain miRNA-binding sites [97]. Furthermore, the coordinated regulatory network of ncRNAs actively participated in the silencing of gene transcription by modulating chromatin modifications, primarily targeted towards TEs and other repetitive gene regions, further influencing the specific gene expression patterns [81]. The characteristic of lncRNAs was the absence of long ORFs (Open Reading Frames), making them suitable precursors for the generation of miRNAs and 21-nt phasiRNAs. The research evidence indicated that photoperiod-sensitive genic male sterility 1 (*Pms1*) locus encoded a lncRNA *PMS1T*, which was targeted for *miR2118*, leading to the production of 21-nt phasiRNAs, which predominantly accumulated in the *PSMS* strains (a line coming from two-line hybrid rice breeding) under long-day conditions [98]. Furthermore, a lncRNA, designated as *LDMAR,* was essential for maintaining normal male fertility in rice plants exposed to extended periods of sunlight. Additionally, a siRNA, known as *Psi-LDMAR* came from the sense strand of the *AK111270* transcript of the *LDMAR* promoter region [99]. The vast majority of phasiRNAs were generated from the 1171 intergenic range, more specifically, within this range, over 700 were lincRNAs, bearing consensus sequences that were complementary to *miR218* that were specifically transcribed in inflorescences. Consequently, it could be deduced that lincRNAs, phasRNAs, and *miR218* participated in the reproductive-specific stage [100]. In addition, an increasing body of research indicated that the factors associated with miRNAs engaged with a broader array of ncRNAs by utilizing to compete for miRNA-binding sites, or by sequestering miRNA transcripts to influence the expression of target mRNAs [101]. In the realm of ceRNA or TMs in experimental and computational identified processes within plants were contained protein-coding RNAs, lncRNAs, viral RNAs, pseudogenes, circRNA species, and artificial RNAs [71]. When it comes to the classification of ceRNAs, artificial RNAs represent a type of engineered artificial target mimics (aTMs) created through the short-tandem target mimicry (STTM) technology apart from naturally occurring endogenous target mimics (eTMs) [102] (Fig. 4).

### ncRNAs drive some functional micropeptides

LncRNAs have conventionally been characterized by their lack of protein-coding potential [25]. However, with the emergence and growing application of techniques such as polysome profiling, techniques of purification, or sequencing of the ribosome, it has become possible that some lncRNAs may indeed encode functional micropeptides due to the presence of some short ORFs within lncRNAs [103]. Moreover, a report detailed the integrated strategies adopted to investigate and characterize the extensive translation of functional micropeptides [104]. Furthermore, lncRNAs translated into micropeptides depended on two regulatory factors located upstream of ORFs, including internal ribosome entry site (IRES) and N6-methyladenosine (m6A) methylation conserved sites. IRES is responsible for recruiting and assembling ribosomes, while m6A methylation is associated with the activation of endogenous ncRNAs translation [105]. In *G.max* and *G.sojae* root tissues, it was revealed that 179 lncRNAs code 153 micropeptides, which were mainly co-expression processes related to coding proteins. These proteins are mainly related to precursors of metabolites and energy, ATP synthesis coupled electronic transmission, light reaction, response to defense, and photosynthesis [106]. In *Physcomitrella patens*, several micropeptides lncRNA-sORFs were identified and participated in growth and development in moss, including *Pp3c9_sORF1544*, *Pp3c18_sORF57*, *Pp3c25_sORF1000*, *Pp3c25_sORF1253* [107]. Moreover, it predicted that most circRNAs had the potential to code for micropeptides. For example, the *hop stunt viroid (HSV)* and *eggplant latent viroid (ELV)* circRNAs had been associated with polysomes, presenting their capacity for translation, which was supported by the existence of several putative ORFs with encoding potential and subcellular localization signals [108]. Pri-miRNAs could encode micropeptides in plants, referred to as *miPEPs*, such as *miPEP17b* from *M. truncatula*, and the small peptide *VVI-miPEP171d1* originated from the first ORF of grapevine *pri-miR171d*. These *miPEPs* often enhance the transcription of respective pri-miRNAs to fulfill regulation roles [104]. In *Arabidopsis*, micropeptide *miPEP858a* and *miPEP156a* are encoded from *pri-miR858a* and *pri-miR156a,* respectively. *miPEP858a* was involved in the phenylpropanoid pathway and plant growth while *miPEP156a* influenced root development [109].

## Conclusion

This review delves into the multifaceted roles of ncRNAs in various aspects of plant growth and response to environmental stimuli. To structure the discussion, first, the ncRNAs are categorized into small RNAs, medium RNAs, lncRNAs, and circle RNAs based on their length and structure form. Furthermore, the review provides a comprehensive exploration of the origin and the mechanism underlying the production of these ncRNAs. Subsequently, the focus shifts to the regulatory mode of lncRNA in proximity to coding genes, which can be situated in various coding gene regions, including promoters, 5′ UTRs, introns, exons, and 3′ UTRs. Additionally, this review highlights the novel roles that lncRNAs as core components can participate in phase separation with the advance of sequence technology. This review also underscores the coordinated regulation involving lncRNAs, miRNAs, and siRNAs based on the pivotal roles of lncRNAs. Finally, as an emerging theme, this review addressed the growing body of evidence that some ncRNAs could code certain function micropeptides, overturning the traditional notion of ncRNAs with non-coding capacity.

Indeed, the ever-mounting evidence of lncRNAs involvement underscores their diverse and influential roles in plant growth and response to environmental stimuli. While certain advanced methods and computational algorithms have been proposed for predicting functional lncRNAs, the subsequent high-cost validation methods can impede the rapid development of our understanding in this field. Consequently, there is a pressing need to explore more efficient and cost-effective approaches for lncRNAs research, of course, the methods originated from animals will be a great inspiration for us.

The interconnected regulation between lncRNAs and other ncRNAs highlights the importance of constructing a comprehensive and coordinated regulation network among these ncRNA types, which is very necessary for us to understand the real function of lncRNAs. Despite the limited sequence conservation of lncRNAs, the rich landscape of ncRNAs as robust regulatory elements significantly augment our understanding of intricate regulatory networks governing plant growth, development, and stress responses. The ever-evolving landscape holds the promise of even more intriguing and uncharted roles for ncRNAs awaiting our exploration.

## Data Availability

Data availability does not apply to this review article as no new data were created or analysed in this study.

## References

[1] Rai MI , Alam M, Lightfoot DA. et al. Classification and experimental identification of plant long non-coding RNAs. Genomics. 2019;111:997–1005 29679643 10.1016/j.ygeno.2018.04.014

[2] Chao HY , Hu Y, Zhao L. et al. Biogenesis, functions, interactions, and resources of non-coding RNAs in plants. Int J Mol Sci. 2022;23:3695 35409060 10.3390/ijms23073695 PMC8998614

[3] Yu Y , Zhang YC, Chen XM. et al. Plant noncoding RNAs: hidden players in development and stress responses. Annu Rev Cell Dev Bi. 2019;35:407–31 10.1146/annurev-cellbio-100818-125218 PMC8034839 31403819

[4] Ben Amor B , Wirth S, Merchan F. et al. Novel long non-protein coding RNAs involved in Arabidopsis differentiation and stress responses. Genome Res. 2009;19:57–69 18997003 10.1101/gr.080275.108 PMC2612962

[5] Axtell MJ . Classification and comparison of small RNAs from plants. Annu Rev Plant Biol. 2013;64:137–59 23330790 10.1146/annurev-arplant-050312-120043

[6] Cuperus JT , Carbonell A, Fahlgren N. et al. Unique functionality of 22-nt miRNAs in triggering RDR6-dependent siRNA biogenesis from target transcripts in. Nat Struct Mol Biol. 2010;17:997–1003 20562854 10.1038/nsmb.1866 PMC2916640

[7] Vazquez F , Blevins T, Ailhas J. et al. Evolution of genes generates novel microRNA classes. Nucleic Acids Res. 2008;36:6429–38 18842626 10.1093/nar/gkn670 PMC2582634

[8] Henderson IR , Zhang X, Lu C. et al. Dissecting *Arabidopsis thaliana* DICER function in small RNA processing, gene silencing and DNA methylation patterning. Nat Genet. 2006;38:721–5 16699516 10.1038/ng1804

[9] Guo R , Chen X, Lin Y. et al. Identification of novel and conserved miRNAs in leaves of in vitro grown *Citrus reticulata* "Lugan" plantlets by Solexa sequencing. Front Plant Sci. 2015;6:1212 26779240 10.3389/fpls.2015.01212 PMC4705231

[10] Rogers K , Chen XM. Biogenesis, turnover, and mode of action of plant MicroRNAs. Plant Cell. 2013;25:2383–99 23881412 10.1105/tpc.113.113159 PMC3753372

[11] Song X , Li Y, Cao X. et al. MicroRNAs and their regulatory roles in plant–environment interactions. Annu Rev Plant Biol. 2019;70:489–525 30848930 10.1146/annurev-arplant-050718-100334

[12] Deleris A , Gallego-Bartolome J, Bao J. et al. Hierarchical action and inhibition of plant dicer-like proteins in antiviral defense. Science. 2006;313:68–71 16741077 10.1126/science.1128214

[13] Li J , Yang Z, Yu B. et al. Methylation protects miRNAs and siRNAs from a 3′-end uridylation activity in Arabidopsis. Curr Biol. 2005;15:1501–7 16111943 10.1016/j.cub.2005.07.029 PMC5127709

[14] Ren G , Xie M, Zhang S. et al. Methylation protects microRNAs from an AGO1-associated activity that uridylates 5′ RNA fragments generated by AGO1 cleavage. Proc Natl Acad Sci U S A. 2014;111:6365–70 24733911 10.1073/pnas.1405083111 PMC4035956

[15] Baldrich P , Beric A, Meyers BC. Despacito: the slow evolutionary changes in plant microRNAs. Curr Opin Plant Biol. 2018;42:16–22 29448158 10.1016/j.pbi.2018.01.007

[16] Debernardi JM , Rodriguez RE, Mecchia MA. et al. Functional specialization of the plant miR396 regulatory network through distinct MicroRNA-target interactions. PLoS Genet. 2012;8:e1002419 22242012 10.1371/journal.pgen.1002419 PMC3252272

[17] Agrawal N , Dasaradhi PVN, Mohmmed A. et al. RNA interference: biology, mechanism, and applications. Microbiol Mol Biol R. 2003;67:657–85 10.1128/MMBR.67.4.657-685.2003 PMC309050 14665679

[18] Cuerda-Gil D , Slotkin RK. Non-canonical RNA-directed DNA methylation. Nat Plants. 2016;2:16163 27808230 10.1038/nplants.2016.163

[19] Liu TT , Zhu D, Chen W. et al. A global identification and analysis of small nucleolar RNAs and possible intermediate-sized non-coding RNAs in Oryza sativa. Mol Plant. 2013;6:830–46 22986792 10.1093/mp/sss087 PMC3716300

[20] Franz W , Witold F. U6 snRNA genes of Arabidopsis are transcribed by RNA polymerase III but contain the same two upstream promoter elements as RNA polymerase II-transcribed U-snRNA genes. Nucleic Acids Res. 1990;18:3451–8 2362802 10.1093/nar/18.12.3451 PMC330996

[21] Wang YQ , Wang X, Deng W. et al. Genomic features and regulatory roles of intermediate-sized non-coding RNAs in Arabidopsis. Mol Plant. 2014;7:514–27 24398630 10.1093/mp/sst177

[22] Wierzbicki AT , Blevins T, Swiezewski S. Long noncoding RNAs in plants. Annu Rev Plant Biol. 2021;72:245–71 33752440 10.1146/annurev-arplant-093020-035446

[23] Marquardt S , Raitskin O, Wu Z. et al. Functional consequences of splicing of the antisense transcript COOLAIR on FLC transcription. Mol Cell. 2014;54:156–65 24725596 10.1016/j.molcel.2014.03.026 PMC3988885

[24] Matzke MA , Mosher RA. RNA-directed DNA methylation: an epigenetic pathway of increasing complexity. Nat Rev Genet 2014;**15**:394. 10.1038/nrg3683 24805120

[25] Ponting CP , Oliver PL, Reik W. Evolution and functions of long noncoding RNAs. Cell. 2009;136:629–41 19239885 10.1016/j.cell.2009.02.006

[26] Jeck WR , Sharpless NE. Detecting and characterizing circular RNAs. Nat Biotechnol. 2014;32:453–61 24811520 10.1038/nbt.2890 PMC4121655

[27] Zhang XO , Dong R, Zhang Y. et al. Diverse alternative back-splicing and alternative splicing landscape of circular RNAs. Genome Res. 2016;26:1277–87 27365365 10.1101/gr.202895.115 PMC5052039

[28] Chen LL , Yang L. Regulation of circRNA biogenesis. RNA Biol. 2015;12:381–8 25746834 10.1080/15476286.2015.1020271 PMC4615371

[29] Lu Z , Grigory SF, Noto JJ. et al. Metazoan tRNA introns generate stable circular RNAs in vivo. RNA. 2015;21:1554–65 26194134 10.1261/rna.052944.115 PMC4536317

[30] Ye CY , Chen L, Liu C. et al. Widespread noncoding circular RNAs in plants. New Phytol. 2015;208:88–95 26204923 10.1111/nph.13585

[31] Lai D , Meyer IM. A comprehensive comparison of general RNA-RNA interaction prediction methods. Nucleic Acids Res. 2016;44:e61 26673718 10.1093/nar/gkv1477 PMC4838349

[32] Conn VM , Hugouvieux V, Nayak A. et al. A circRNA from SEPALLATA3 regulates splicing of its cognate mRNA through R-loop formation. Nat Plants. 2017;3:17053 28418376 10.1038/nplants.2017.53

[33] Tang BH , Hao ZQ, Zhu YF. et al. Genome-wide identification and functional analysis of circRNAs in Zea mays. PLoS One. 2018;13:e0202375 30533052 10.1371/journal.pone.0202375 PMC6289457

[34] Tan JJ , Zhou ZJ, Niu YJ. et al. Identification and functional characterization of tomato CircRNAs derived from genes involved in fruit pigment accumulation. Sci Rep-Uk. 2017;7:8594 10.1038/s41598-017-08806-0 PMC5561264 28819222

[35] Chen LL . The biogenesis and emerging roles of circular RNAs. Nat Rev Mol Cell Bio. 2016;17:205–11 26908011 10.1038/nrm.2015.32

[36] Thomas QA , Ard R, Liu J. et al. Transcript isoform sequencing reveals widespread promoter-proximal transcriptional termination in Arabidopsis. Nat Commun. 2020;11:2589 32444691 10.1038/s41467-020-16390-7 PMC7244574

[37] Seo JS , Diloknawarit P, Park BS. et al. ELF18-INDUCED LONG NONCODING RNA 1 evicts fibrillarin from mediator subunit to enhance PATHOGENESIS-RELATED GENE 1 (PR1) expression. New Phytol. 2019;221:2067–79 30307032 10.1111/nph.15530

[38] Ariel F , Teddy J, Latrasse D. et al. Noncoding transcription by alternative RNA polymerases dynamically regulates an auxin-driven chromatin loop. Molcular Cell. 2014;55:383–96 10.1016/j.molcel.2014.06.011 25018019

[39] Moison M , Pacheco JM, Lucero L. et al. The lncRNA interacts with the transcription factor WRKY42 to trigger root hair cell expansion in response to cold. Mol Plant. 2021;14:937–48 33689931 10.1016/j.molp.2021.03.008

[40] Kim DH , Sung S. Vernalization-triggered intragenic chromatin loop formation by long noncoding RNAs. Dev Cell. 2017;40:302–312.e4 28132848 10.1016/j.devcel.2016.12.021 PMC5303624

[41] Li Q , Gent JI, Zynda G. et al. RNA-directed DNA methylation enforces boundaries between heterochromatin and euchromatin in the maize genome. Proc Natl Acad Sci U S A. 2015;112:14728–33 26553984 10.1073/pnas.1514680112 PMC4664327

[42] Wang H , Chung PJ, Liu J. et al. Genome-wide identification of long noncoding natural antisense transcripts and their responses to light in Arabidopsis. Genome Res. 2014;24:444–53 24402519 10.1101/gr.165555.113 PMC3941109

[43] Zhu T , Yang C, Xie Y. et al. Shade-induced lncRNA PUAR promotes shade response by repressing PHYA expression. EMBO Rep. 2023;24:e56105 36970931 10.15252/embr.202256105 PMC10157314

[44] Ye X , Wang S, Zhao X. et al. Role of lncRNAs in cis- and trans-regulatory responses to salt in *Populus trichocarpa*. Plant J. 2022;110:978–93 35218100 10.1111/tpj.15714

[45] Pumplin N , Sarazin A, Jullien PE. et al. DNA methylation influences the expression of isoforms, which encode proteins of alternative localization and function. Plant Cell. 2016;28:2786–804 27956586 10.1105/tpc.16.00554 PMC5155348

[46] Wu HW , Deng S, Xu H. et al. A noncoding RNA transcribed from the AGAMOUS (AG) second intron binds to CURLY LEAF and represses AG expression in leaves. New Phytol. 2018;219:1480–91 29862530 10.1111/nph.15231

[47] Shin WJ , Nam AH, Kim JY. et al. Intronic long noncoding RNA, RICE FLOWERING ASSOCIATED (RIFLA) regulates OsMADS56-mediated flowering in rice. Plant Sci. 2022;320:111278 35643617 10.1016/j.plantsci.2022.111278

[48] Hammond MC , Wachter A, Breaker RR. A plant 5S ribosomal RNA mimic regulates alternative splicing of transcription factor IIIA pre-mRNAs. Nat Struct Mol Biol. 2009;16:541–9 19377483 10.1038/nsmb.1588 PMC2680232

[49] Ai G , Li T, Zhu H. et al. BPL3 binds the long non-coding RNA nalncFL7 to suppress FORKED-LIKE7 and modulate HAI1-mediated MPK3/6 dephosphorylation in plant immunity. Plant Cell. 2023;35:598–616 36269178 10.1093/plcell/koac311 PMC9806616

[50] Chen W , Zhu T, Shi Y. et al. An antisense intragenic lncRNA SEAIRa mediates transcriptional and epigenetic repression of SERRATE in Arabidopsis. Proc Natl Acad Sci U S A. 2023;120:e2216062120 36857348 10.1073/pnas.2216062120 PMC10013867

[51] Krzyszton M , Zakrzewska-Placzek M, Kwasnik A. et al. Defective XRN3-mediated transcription termination in Arabidopsis affects the expression of protein-coding genes. Plant J. 2018;93:1017–31 29356198 10.1111/tpj.13826

[52] Kindgren P , Ard R, Ivanov M. et al. Transcriptional read-through of the long non-coding RNA SVALKA governs plant cold acclimation. Nat Commun. 2018;9:4561 30385760 10.1038/s41467-018-07010-6 PMC6212407

[53] Xu W , Xu H, Li K. et al. The R-loop is a common chromatin feature of the Arabidopsis genome. Nat Plants. 2017;3:704–14 28848233 10.1038/s41477-017-0004-x

[54] Hisanaga T , Okahashi K, Yamaoka S. et al. A *cis*-acting bidirectional transcription switch controls sexual dimorphism in the liverwort. EMBO J. 2019;38:e100240 30609993 10.15252/embj.2018100240 PMC6418429

[55] Liu NK , Xu Y, Li Q. et al. A lncRNA fine-tunes salicylic acid biosynthesis to balance plant immunity and growth. Cell Host Microbe. 2022;30:1124–38 35908550 10.1016/j.chom.2022.07.001

[56] Zhu BZ , Yang Y, Li R. et al. RNA sequencing and functional analysis implicate the regulatory role of long non-coding RNAs in tomato fruit ripening. J Exp Bot. 2015;66:4483–95 25948705 10.1093/jxb/erv203 PMC4507755

[57] Yu TT , Tzeng DTW, Li R. et al. Genome-wide identification of long non-coding RNA targets of the tomato MADS box transcription factor RIN and function analysis. Ann Bot-London. 2019;123:469–82 10.1093/aob/mcy178 PMC6377105 30376036

[58] Li R , Fu DQ, Zhu BZ. et al. CRISPR/Cas9-mediated mutagenesis of lncRNA1459 alters tomato fruit ripening. Plant J. 2018;94:513–24 29446503 10.1111/tpj.13872

[59] Yang J , He R, Qu Z. et al. Long noncoding RNA ARTA controls ABA response through MYB7 nuclear trafficking in Arabidopsis. Dev Cell. 2023;58:1206–1217.e4 37290444 10.1016/j.devcel.2023.05.003

[60] Zhou YF , Zhang YC, Sun YM. et al. The parent-of-origin lncRNA regulates rice endosperm development. Nat Commun. 2021;12:6525 34764271 10.1038/s41467-021-26795-7 PMC8585977

[61] Xiao Y , Kang B, Li M. et al. Transcription of lncRNA ACoS-AS1 is essential to trans-splicing between SlPsy1 and ACoS-AS1 that causes yellow fruit in tomato. RNA Biol. 2020;17:596–607 31983318 10.1080/15476286.2020.1721095 PMC7237131

[62] Yang ZC , Yang Z, Yang C. et al. Identification and genetic analysis of alternative splicing of long non-coding RNAs in tomato initial flowering stage. Genomics. 2020;112:897–907 31175976 10.1016/j.ygeno.2019.06.005

[63] Rigo R , Bazin J, Romero-Barrios N. et al. The Arabidopsis lncRNA ASCO modulates the transcriptome through interaction with splicing factors. EMBO Rep. 2020;21:e48977 32285620 10.15252/embr.201948977 PMC7202219

[64] Xie XD , Jin J, Wang C. et al. Investigating nicotine pathway-related long non-coding RNAs in tobacco. Front Genet. 2023;13:1102183 36744176 10.3389/fgene.2022.1102183 PMC9892058

[65] Kosmacz M , Gorka M, Schmidt S. et al. Protein and metabolite composition of Arabidopsis stress granules. New Phytol. 2019;222:1420–33 30664249 10.1111/nph.15690

[66] Emenecker RJ , Holehouse AS, Strader LC. Emerging roles for phase separation in plants. Dev Cell. 2020;55:69–83 33049212 10.1016/j.devcel.2020.09.010 PMC7577370

[67] Khong A , Matheny T, Jain S. et al. The stress granule transcriptome reveals principles of mRNA accumulation in stress granules. Mol Cell. 2017;68:808–820.e5 29129640 10.1016/j.molcel.2017.10.015 PMC5728175

[68] Kearly A , Nelson ADL, Skirycz A. et al. Composition and function of stress granules and P-bodies in plants. Semin Cell Dev Biol. 2022;156:167–75 36464613 10.1016/j.semcdb.2022.11.008

[69] Pitchiaya S , Mourao MDA, Jalihal AP. et al. Dynamic recruitment of single RNAs to processing bodies depends on RNA functionality. Mol Cell. 2019;74:521–533.e6 30952514 10.1016/j.molcel.2019.03.001 PMC6499680

[70] Ariel F , Jegu T, Latrasse D. et al. Noncoding transcription by alternative RNA polymerases dynamically regulates an auxin-driven chromatin loop. Mol Cell. 2014;55:383–96 25018019 10.1016/j.molcel.2014.06.011

[71] Thomson DW , Dinger ME. Endogenous microRNA sponges: evidence and controversy. Nat Rev Genet. 2016;17:272–83 27040487 10.1038/nrg.2016.20

[72] Li YJ , Li XR, Yang J. et al. Natural antisense transcripts of MIR398 genes suppress microR398 processing and attenuate plant thermotolerance. Nat Commun. 2020;11:5351 33093449 10.1038/s41467-020-19186-x PMC7582911

[73] Salmena L , Poliseno L, Tay Y. et al. A ceRNA hypothesis: the Rosetta stone of a hidden RNA language? Cell. 2011;146:353–8 21802130 10.1016/j.cell.2011.07.014 PMC3235919

[74] Franco-Zorrilla JM , Valli A, Todesco M. et al. Target mimicry provides a new mechanism for regulation of microRNA activity. Nat Genet. 2007;39:1033–7 17643101 10.1038/ng2079

[75] Hou X , Cui J, Liu W. et al. LncRNA39026 enhances tomato resistance to *Phytophthora infestans* by decoying miR168a and inducing PR gene expression. Phytopathology. 2019;110:873–80 10.1094/PHYTO-12-19-0445-R 31876247

[76] Sun Z , Huang K, Han Z. et al. Author correction: genome-wide identification of Arabidopsis long noncoding RNAs in response to the blue light. Sci Rep-Uk. 2020;10:11446 10.1038/s41598-020-68529-7 PMC7338447 32632212

[77] Jiang N , Cui J, Shi Y. et al. Tomato lncRNA23468 functions as a competing endogenous RNA to modulate NBS-LRR genes by decoying miR482b in the tomato-Phytophthora infestans interaction. Hortic Res. 2019;6:28 30729018 10.1038/s41438-018-0096-0 PMC6355781

[78] Wang MJ , Yuan D, Tu L. et al. Long noncoding RNAs and their proposed functions in fibre development of cotton (Gossypium spp.). New Phytol. 2015;207:1181–97 25919642 10.1111/nph.13429

[79] Zhang YC , Yu Y, Wang CY. et al. Overexpression of microRNA OsmiR397 improves rice yield by increasing grain size and promoting panicle branching. Nat Biotechnol. 2013;31:848–52 23873084 10.1038/nbt.2646

[80] Xin MM , Wang Y, Yao Y. et al. Identification and characterization of wheat long non-protein coding RNAs responsive to powdery mildew infection and heat stress by using microarray analysis and SBS sequencing. BMC Plant Biol. 2011;11:61 10.1186/1471-2229-11-61 PMC3079642 21473757

[81] Zheng Q , Rowley MJ, Böhmdorfer G. et al. RNA polymerase V targets transcriptional silencing components to promoters of protein-coding genes. Plant J. 2013;73:179–89 23013441 10.1111/tpj.12034 PMC5096367

[82] McKinlay A , Podicheti R, Wendte JM. et al. RNA polymerases IV and V influence the 3 boundaries of polymerase II transcription units in Arabidopsis. RNA Biol. 2018;15:269–79 29199514 10.1080/15476286.2017.1409930 PMC5798951

[83] Borsani O , Zhu JH, Verslues PE. et al. Endogenous siRNAs derived from a pair of natural cis-antisense transcripts regulate salt tolerance in Arabidopsis. Cell. 2005;123:1279–91 16377568 10.1016/j.cell.2005.11.035 PMC3137516

[84] Li S , Yu X, Lei N. et al. Genome-wide identification and functional prediction of cold and/or drought-responsive lncRNAs in cassava. Sci Rep. 2017;7:45981 28387315 10.1038/srep45981 PMC5384091

[85] Xia J , Zeng C, Chen Z. et al. Endogenous small-noncoding RNAs and their roles in chilling response and stress acclimation in Cassava. BMC Genomics. 2014;15:634 25070534 10.1186/1471-2164-15-634 PMC4124141

[86] Yang YW , Liu T, Shen D. et al. Tomato yellow leaf curl virus intergenic siRNAs target a host long noncoding RNA to modulate disease symptoms. PLoS Pathog. 2019;15:e1007534 30668603 10.1371/journal.ppat.1007534 PMC6366713

[87] Zilberman D , Cao XF, Jacobsen SE. ARGONAUTE4 control of locus-specific siRNA accumulation and DNA and histone methylation. Science. 2003;299:716–9 12522258 10.1126/science.1079695

[88] Qi YJ , He X, Wang XJ. et al. Distinct catalytic and non-catalytic roles of ARGONAUTE4 in RNA-directed DNA methylation. Nature. 2006;443:1008–12 16998468 10.1038/nature05198

[89] Wierzbicki AT , Ream TS, Haag JR. et al. RNA polymerase V transcription guides ARGONAUTE4 to chromatin. Nat Genet. 2009;41:630–4 19377477 10.1038/ng.365 PMC2674513

[90] Rothi MH , Tsuzuki M, Sethuraman S. et al. Reinforcement of transcriptional silencing by a positive feedback between DNA methylation and non-coding transcription. Nucleic Acids Res. 2021;49:9799–808 34469565 10.1093/nar/gkab746 PMC8464056

[91] Matzke M , Kanno T, Daxinger L. et al. RNA-mediated chromatin-based silencing in plants. Curr Opin Cell Biol. 2009;21:367–76 19243928 10.1016/j.ceb.2009.01.025

[92] Li JZ , Li N, Zhu L. et al. Mutation of a major CG methylase alters genome-wide lncRNA expression in rice. G3. 2021;11:jkab049 33617633 10.1093/g3journal/jkab049 PMC8049413

[93] Lu X , Wang X, Chen X. et al. Single-base resolution methylomes of upland cotton (*Gossypium hirsutum* L.) reveal epigenome modifications in response to drought stress. BMC Genomics. 2017;18:297 28407801 10.1186/s12864-017-3681-y PMC5390369

[94] Zhang PJ , Meng X, Chen H. et al. PlantCircNet: a database for plant circRNA-miRNA-mRNA regulatory networks. Database-Oxford. 2017;2017:bax089 31725858 10.1093/database/bax089 PMC5727401

[95] Macovei A , Tuteja N. microRNAs targeting DEAD-box helicases are involved in salinity stress response in rice (Oryza sativa L.). BMC Plant Biol. 2012;12:183 23043463 10.1186/1471-2229-12-183 PMC3502329

[96] Guleria P , Yadav SK. Identification of miR414 and expression analysis of conserved miRNAs from Stevia rebaudiana. Genom Proteom Bioinform. 2011;9:211–7 10.1016/S1672-0229(11)60024-7 PMC5054151 22289477

[97] Zuo JH , Wang Q, Zhu BZ. et al. Deciphering the roles of circRNAs on chilling injury in tomato. Biochem Biophys Res Commun. 2016;479:132–8 27402275 10.1016/j.bbrc.2016.07.032

[98] Fan YR , Yang J, Mathioni SM. et al. PMS1T, producing phased small-interfering RNAs, regulates photoperiod-sensitive male sterility in rice. Proc Natl Acad Sci U S A. 2016;113:15144–9 27965387 10.1073/pnas.1619159114 PMC5206514

[99] Ding JH , Shen J, Mao H. et al. RNA-directed DNA methylation is involved in regulating photoperiod-sensitive male sterility in rice. Mol Plant. 2012;5:1210–6 23024213 10.1093/mp/sss095

[100] Komiya R , Ohyanagi H, Niihama M. et al. Rice germline-specific Argonaute MEL1 protein binds to phasiRNAs generated from more than 700 lincRNAs. Plant J. 2014;78:385–97 24635777 10.1111/tpj.12483

[101] Paschoal AR , Lozada-Chavez I, Domingues DS. et al. ceRNAs in plants: computational approaches and associated challenges for target mimic research. Brief Bioinform. 2018;19:1273–89 28575144 10.1093/bib/bbx058

[102] Zand Karimi H , Innes RW. Molecular mechanisms underlying host-induced gene silencing. Plant Cell. 2022;34:3183–99 35666177 10.1093/plcell/koac165 PMC9421479

[103] Ruiz-Orera J , Messeguer X, Subirana JA. et al. Long non coding RNAs as a source of new peptides. elife. 2014;3:1–24 10.7554/eLife.03523 PMC4359382 25233276

[104] Chen YJ , Li D, Fan W. et al. PsORF: a database of small ORFs in plants. Plant Biotechnol J. 2020;18:2158–60 32333496 10.1111/pbi.13389 PMC7589237

[105] Yang YB , Gao X, Zhang M. et al. Novel role of FBXW7 circular RNA in repressing Glioma tumorigenesis. Jnci-J Natl Cancer I. 2018;110:304–15 10.1093/jnci/djx166 PMC6019044 28903484

[106] Lin X , Lin W, Ku YS. et al. Analysis of soybean long non-coding RNAs reveals a subset of small peptide-coding transcripts(1[OPEN]). Plant Physiol. 2020;182:1359–74 31882456 10.1104/pp.19.01324 PMC7054870

[107] Fesenko I , Kirov I, Kniazev A. et al. Distinct types of short open reading frames are translated in plant cells. Genome Res. 2019;29:1464–77 31387879 10.1101/gr.253302.119 PMC6724668

[108] Marquez-Molins J , Navarro JA, Seco LC. et al. Might exogenous circular RNAs act as protein-coding transcripts in plants? RNA Biol. 2021;18:98–107 34392787 10.1080/15476286.2021.1962670 PMC8677015

[109] Sharma A , Badola PK, Bhatia C. et al. Primary transcript of miR858 encodes regulatory peptide and controls flavonoid biosynthesis and development in Arabidopsis. Nat Plants. 2020;6:1262–74 32958895 10.1038/s41477-020-00769-x

